# Site engagement in implementation research: Introducing SEAMLIS as a conceptual and measurement framework

**DOI:** 10.1186/s40352-025-00349-1

**Published:** 2025-07-05

**Authors:** Sarah D. Jones, John P. Bartkowski, Steven Belenko, Jennifer E. Becan, Faye S. Taxman, Gail A. Wasserman, Gregory A. Aarons, Larkin S. McReynolds, Cheyenne Dolbear, Xiaohe Xu

**Affiliations:** 1 Temple University, Philadelphia, USA; 2 The University of Texas at San Antonio, San Antonio, USA; 3 Texas Christian University, Fort Worth, USA; 4 George Mason University, Fairfax, USA; 5 Columbia University, New York, USA; 6 University of California, San Diego, San Diego, USA

**Keywords:** Implementation science, Intervention, Site engagement, Justice agencies, Health, Measurement, SEAMLIS

## Abstract

**Background:**

Multisite implementation research in justice and health settings often does not systematically assess differential degrees of project involvement among participating sites, despite its implications for both research and the intervention. Tracking organization and participant involvement across sites, when attempted, has typically entailed the use of discrete and sometimes disjointed fidelity measures that may not accurately reflect engagement with a project. This article advances a more comprehensive and sophisticated conceptual model for measuring and monitoring site engagement. This conceptual model was developed from a literature review of the implementation science and related disciplines while being informed by multisite project implementation experience. We propose the Site Engagement Activity Model Leveraging Implementation Science (SEAMLIS), a conceptual model that holistically identifies the breadth of agency participation (diverse activities such as trainings, meetings, etc.) and duration of site engagement (participation levels from inception to completion) to be measured, assessed, and reported.

**Case presentation:**

We also describe Juvenile Justice Translational Research on Interventions for Adolescents in the Legal System (JJ-TRIALS), a 36-site implementation research project, as an illustrative case example of our proposed model. We then operationalize all proposed domains and subdomains and specify key measures from the project.

**Conclusions:**

We provide analytical recommendations for the application and future research of the proposed model in health and justice settings. In multisite implementation research, site engagement could be fruitfully used as an independent, dependent, or intervening (moderating or mediating) variable.

**Trial registration:**

NCT02672150, February 3, 2016.

## Background

The emergence of an implementation science perspective in criminal justice is relatively recent compared to other fields (Taxman, 2025; Taxman & Belenko, 2025), yet it is arguably worth increased attention given the unique challenges posed by justice settings (e.g., focus on control and punishment; Zielinski et al., 2020) and the needs of the incarcerated population (Van Deinse et al., 2023). In their systematic review of the application of implementation science in correctional health interventions, Van Deinse et al. (2023) found that implementation research predominantly focuses on the examination of three areas of focus: implementation determinants, outcomes, and strategies. However, largely unrecognized in the literature is the highly consequential factor of site engagement over the course of the implementation process.

Lessons from multisite efforts to study the implementation of EBPs (e.g., Belenko et al., 2013; Belenko et al., 2022; Bunger et al., 2024; Chamberlain et al., 2011; Knight et al., 2022; Panzano & Roth, 2006) indicate that successful implementation of EBPs is inconsistent and can vary widely across sites and agencies. Because the implementation of EBPs and the shift in routinized practice takes time and effort, it is reasonable to presume that the implementing agency and/or organization’s level of involvement may fluctuate over the course of the implementation effort. Yet, a comprehensive and systematic understanding of the dynamic construct of “site engagement” is lacking in the field of implementation science. With limited exception, to our knowledge, very few sources contend with the concept directly (Goodlett et al., 2020; Wells et al., 2018), and none with a focus on the criminal justice system. Differences in site engagement can yield differences in implementation, intervention, and health service outcomes and can affect the degree to which a site yields similar outcomes as in the research literature.

The purpose of this paper is to fill this gap in the literature by developing a conceptual model for site engagement. Informed by a thorough review of multidisciplinary literatures, we propose the Site Engagement Activity Model Leveraging Implementation Science (SEAMLIS), which includes four key site engagement domains: *Responsiveness*, *Collaboration*, *Feedback*, and *Support Exchange*. We then illustrate the model using the large 36-site implementation research project, Juvenile Justice Translational Research on Interventions for Adolescents in the Legal System (JJ-TRIALS), funded by the National Institute on Drug Abuse (Knight et al., 2015), including the observed variation in site engagement across the project’s original 36 sites. We also specify activity measures from the project to operationalize the proposed domains and subdomains. To close, we discuss potential applications and limitations of the model and corresponding directions for future research. This paper aims to offer a preliminary and systematic conceptualization and operationalization of site engagement both broadly and specifically in the context of justice system agencies.

### Introduction to site engagement

The use of multisite designs in EBP implementation research has become increasingly prevalent, particularly in the health, justice, and social services sectors (e.g., Becan et al., 2018; Hunter et al., 2020; Kim et al., 2020; Watson et al., 2020). However, variation among sites in the degree to which they participate or are involved in such research, as well as variation in implementation outcomes, is rarely addressed or conceptualized systematically. Despite this gap, site engagement remains critical to implementation science given its consequential nature for research, intervention, and health outcomes for justice populations. For example, justice system agencies that exhibit low engagement in the process of implementing a treatment program for persons with substance use disorder may detrimentally impact recovery and other client outcomes. Given its import, efforts to better understand site engagement both conceptually and empirically are warranted.

In the context of clinical trials in the medical field, Goodlett et al. (2020, p. 1) define site engagement as the “engagement of clinicians and research teams” and note the importance of understanding variation in the construct because of its potential to “dramatically impact participant accrual and retention, data compliance, and even internal validity of research findings.” Although Goodlett and colleagues do not explicitly define a framework through which to systematically understand and assess site engagement, as we seek to do so in this paper, they do propose numerous best practices on the part of the research team for promoting site engagement within multisite clinical trials (see below). Aside from this initial conceptual exploration, in their systematic review of quality improvement collaboratives, Wells et al. (2018) discuss how key measures of site engagement, such as team participation and submitted improvement plans, were seldom included. Ultimately, site engagement remains a largely under-analyzed component of the broader implementation science literature.

Attempts to track variations in implementation adherence, one possible proxy for site engagement, are sometimes addressed through basic measures of fidelity (see, e.g., Bos et al., 2023; Hill & Erickson, 2019; Rojas-Andrade & Bahamondes, 2019 for reviews), or the degree to which an EBP or intervention is implemented as originally intended (Dusenbury et al., 2003). Fidelity models often adopt a checklist approach or binary measures to determine the completion of essential activities related to the EBP or implementation strategy, focusing on intervention delivery outcomes (Carroll et al., 2007; Dusenbury et al., 2003). Intervention fidelity is typically focused more on EBP adherence to ensure that an intervention is standardized across sites, rather than measuring site cooperation and/or enthusiasm in the implementation process itself. Yet, the growing importance of implementation science suggests a need for the development of a site engagement model that moves beyond fidelity and simplistic measures of adherence (Austin et al., 2024).

To fill this gap in the literature, we propose that site engagement should be conceptualized in a more comprehensive and theoretically informed fashion, thereby facilitating analysis of successful and sustained versus unsuccessful and episodic implementation efforts across sites. We define site engagement as *the degree to which a site exhibits active involvement in diverse facets of a program/intervention over time and supports the goals of a corresponding research study*. We characterize a “site” as an agency, organization, or service provider for which staff and/or leadership are formally participating and providing data for an implementation research study. Our proposed model is informed by previous research and is grounded in a large multisite implementation project that utilized the Exploration-Preparation-Implementation-Sustainment (EPIS) framework (Aarons et al., 2011; Becan et al., 2018; Knight et al., 2015) and aims to push the field beyond its current bounds. To develop this model, we conducted a broad multidisciplinary search of the literature. First, we reviewed the literature on concepts adjacent to site engagement (site/implementation commitment, involvement, etc.) within the field of implementation science. From there, the key domains of site engagement proposed below were thematically developed. Next, we performed a secondary search of the literature beyond the field of implementation science, more targeted to the emergent domains. We further thematically distilled relevant sub-domains for each site engagement domain from this secondary, more multidisciplinary review.

Site engagement can be perceived as an initial activity that precedes implementation or as an overarching process that spans the life of a research study. SEAMLIS is focused holistically on overall project involvement as a multidimensional and variable construct, considering both implementation intervention-based activities and non-intervention factors (e.g., relationship management) that can rise and fall throughout a project. SEAMLIS is based on distinct yet related domains of engagement that can be measured at any time (e.g., study initiation, study completion, or anywhere in between). For example, SEAMLIS can be paired with EPIS or another temporally focused model to examine the peaks and troughs of involvement in the intervention/EBP over the full life of an intervention. Thus, SEAMLIS is attuned to various types of study involvement (e.g., internal functioning, external collaborations) but is also flexible enough to be used in tandem with existing frameworks and models for a more holistic analysis.

### Proposed site engagement domains

**Fig. 1:**
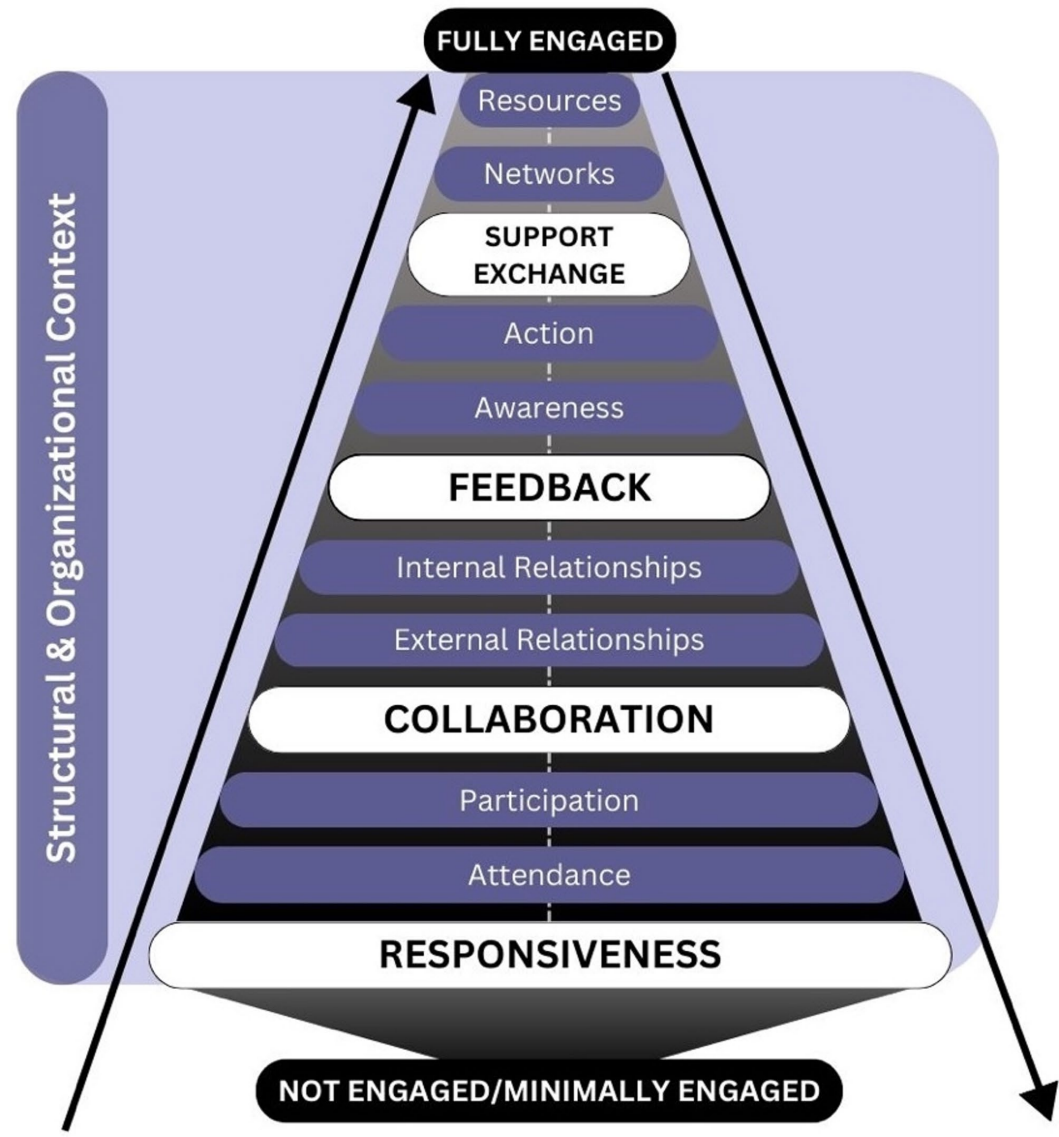
The Site Engagement Activity Model Leveraging Implementation Science (SEAMLIS) Ladder

SEAMLIS defines site engagement as a dynamic attribute. A site may become more involved as implementation proceeds, thereby moving up through the proposed domains, or, conversely, a site may pull back from the implementation process with reduced engagement. Our site engagement model (Fig. 1) proposes four core domains, each comprised of two subdomains for which the first indicates basic involvement and the second indicates the optimal level of involvement. The basic-level subdomain, where the site is involved and following study protocols, is necessary for participation but likely not sufficient for a site to be considered *engaged*. The optimal-level subdomain goes beyond fidelity and represents involvement likely to predict successful and sustainable implementation. Progress throughout the SEAMLIS model can be assessed using various proposed scales and measures, as discussed below.

Upward movement across the conceptual model domains represents a “ladder” to greater site engagement. Although these domains are conceptually distinct, they overlap empirically in their potential operationalization. At study inception, a site may begin its involvement with researchers as part of the initial *Responsiveness* domain. Depending on the quality of that engagement, a site is then classified as being within either the basic- or optimal-level subdomain. To move into the optimal-level subdomain, the site must have met and exceeded the basic-level subdomain classification. As the study progresses, a site may further its involvement in the implementation process and thus move up the pyramid of site engagement into a higher-level domain. Importantly, other structural or organizational factors outside of a site’s control may play significant roles in hindering and/or facilitating implementation, thereby impacting the degree of site engagement.

#### Domain 1: Responsiveness

The first site engagement domain is *Responsiveness*, which is largely operationalized by involvement in internal or inter-organizational events, meetings, and/or trainings. This domain is a basic level of site engagement with two subdomains, Attendance and Participation. Attendance as a subdomain may indicate implementation strategy fidelity, where the site engages in the aforementioned checklist approach. Much like the broader conceptual model, the first subdomain is a building block for the second. Attendance is the basic-level subdomain, and Participation reflects a more actively involved site that demonstrates a higher level of engagement.

##### Attendance subdomain (basic level)

The degree of involvement has been critical to agency partnerships. In a study of organizational commitment to implementation, Margerum (2001) created a typology of organizational participation comprising four core classifications: nonparticipant, observer, partner, and sponsor. Nonparticipants either did not participate at all or were only marginally involved. Observers were those organizations that participated but often watched other organizations and did not make decisions on their own. Other classifications included partner organizations that were actively involved in and supportive of planning and implementation, and sponsor organizations that either held considerable authority or provided the majority of funding and resources for the initiative. The proposed *Responsiveness* domain emphasizes a similar distinction where key site staff must at least observe or attend relevant meetings to be considered a participant and responsive. In an evaluation of quality improvement collaboratives, Rohweder et al. (2019) measured engagement as attendance in collaborative trainings and meetings, finding that engagement declined over time. At an organizational level, to be considered basically engaged, a site must be present at relevant planning meetings and other introductory forums.

##### Participation subdomain (optimal level)

The optimal-level subdomain, Participation, is informed by broader public health literature on community participation, or involvement in activities and discussions to support change and partake in an intervention (Haldane et al., 2019). Findings suggest that responsibility exercised by community members results in increased participation, empowerment, and satisfaction (Butterfoss, 2006). In the early phases, Goodlett et al. (2020) posit that active communication with sites while eliciting their input builds mutual relationships that service the entire project timeline. This subdomain goes beyond simple presence at events or meetings, capturing more involved action that may demonstrate greater commitment that leads to sustained innovation.

#### Domain 2: Collaboration

The next domain in SEAMLIS focuses on *Collaboration* in both the inner and outer settings of an agency, typically demonstrated by strong relationships among researchers, supportive site leadership, and robust team member coordination. Besides EBP or implementation strategy fidelity, this domain reflects a site’s buy-in to the implementation process. It considers the bidirectionality of support and, more generally, engagement. Researchers can play a critical role in empowering sites to pursue and engage in implementation efforts, particularly through engagement in a particular strategy such as training, quality improvement processes, and data-decision making (see Powell et al. (2016) for a list of implementation strategies). Additionally, the support that frontline practitioners receive from supervisors and other staff keeps the agency engaged in the implementation process. Without this critical support, the organization’s staff is likely to face implementation barriers that could thwart successful program adoption (Gumusluoglu & Ilsev, 2009; Scott & Bruce, 1994).

##### External relationships subdomain (basic level)

External Relationships refer to partnerships that a site has with other agencies, typically referred to as the outer setting. This can include researchers guiding the implementation study or a specific protocol. Goodlett et al. (2020) underscore the importance of the researcher’s involvement with site teams early in the trial or, by our extension, during initial implementation as a means to support sites in the change process. The quality of these relationships influences implementation and engagement. By definition, an engaged site supports implementation goals, demonstrating a positive at best or neutral at minimum association with researchers. Importantly, these relationships are bidirectional, and the research team can affect the site’s involvement. Chang (2007) explains the importance of relationship-building in consumer markets and summarizes previous research on numerous dimensions of relationship quality (satisfaction, trust, etc.). We proport that sites that perceive their external relationships to be of high quality are often more engaged and committed to the implementation process.

##### Internal relationships subdomain (optimal level)

The Internal Relationships (within the inner setting) construct recognizes the importance of a well-run and committed organization and focuses on support and involvement among management and staff. Leaders can ensure that implementation remains a priority throughout an agency (Aarons et al., 2014a), and it is necessary for supportive leaders to promote engagement (Darwish et al., 2020). Previous research affirms this pattern, suggesting that supportive leadership is necessary for successful implementation (Aarons et al., 2009a). One factor that separated organizations into the previously described participation typology was organizational guidance and training (Margerum, 2001), where organizations that offer more guidance and better training in the practices are more likely to be actively engaged and supportive of inter-organizational partners. Without the guidance of leaders, organizations often become observers or non-participants; frontline staff may lack the authority to make decisions and promote organizational engagement. For example, a recent cluster randomized trial of the Leadership and Organizational Change for Implementation (LOCI) strategy found that improving leaders’ behaviors along with organizational implementation climate development resulted in moderate to large effect sizes for measurement-based care fidelity and client outcomes (Williams et al., 2023).

This second subdomain of *Collaboration* also involves relationships with and amongst frontline staff. Implementing EBPs is often a team project and requires the support of leadership as well as other team members. When organizational learning is team-based, the acquisition of new knowledge and skills is often more successful and provides continued motivation (Henderson et al., 2014). Individual learning is more difficult to encourage and may fail to serve overall implementation as some organization members may not take an active role. Staff turnover is another threat to the implementation process, requiring the restarting of such processes. For example, EBP implementation efforts involving fidelity monitoring and supportive coaching were protective factors against turnover where the EBP was a good fit for the service system, providers, and clients (Aarons et al., 2009b). In the same study, implementation efforts that excluded fidelity monitoring and supportive coaching were associated with the highest staff turnover, suggesting that staff members may feel unsupported and alienated when EBPs are implemented without sufficient feedback and support.

Similarly, ineffective leadership can negatively impact implementation. The actions of supervisors have been found to be a formidable barrier to successful implementation, including failure to develop an action plan for implementation or lack of role clarity, which leaves frontline practitioners to figure out their roles (Henderson & Douglas, 2022; Rapp et al., 2010). When first-level leaders and middle managers support implementation and efficiency, organizational outcomes are more likely to be positive and lead to successful implementation (Birken et al., 2018). However, if middle managers are not supportive, implementation can be hindered by a lack of information-sharing or staff difficulty in carrying out implementation activities. Therefore, it is important to understand alignment across the structure of organizations implementing an EBP and to be prepared to ensure that leadership at multiple levels is supportive of the process (Byeon et al., 2023).

These studies underscore how implementation is a collaborative and active process requiring the support of all levels of leadership and frontline staff. Without these components, site engagement is likely to be low and implementation may remain a surface-level activity or fail altogether.

#### Domain 3: Feedback

The next domain is *Feedback*, encompassing evaluation of progress or areas of improvement and site-initiated modification informed by assessment. More engaged sites are more likely to pursue tasks independently or contact other agencies in their network to find solutions. This domain largely demonstrates whether a site has reflected on and assessed the implementation process and has taken steps to address its needs. Sites may use evaluation techniques such as data-driven decision-making and performance feedback to monitor performance during the implementation process. *Feedback* builds on the *Collaboration* domain and suggests that more engaged sites take the further step of amending the implementation process as necessary for their specific organizational contexts. Early work supports this conceptualization, demonstrating that evaluation promotes participation within programming (Crump et al., 1996).

##### Awareness subdomain (basic level)

Awareness is achieved when a site indicates its needs or areas for improvement, often at the request of oversight organizations or researchers. Techniques of soliciting feedback and assessing the needs of sites are common in implementation research, and tools for doing so present a critical opportunity for staff or other actors to identify both barriers and facilitators in the implementation process (Geerligs et al., 2018). Additionally, this process is an important opportunity for research teams to facilitate discussion and knowledge sharing in a way that values the site and promotes commitment to implementation (Goodlett et al., 2020). At the basic level, this subdomain demonstrates response to needs assessment measures and thus sets the stage for future responses to feedback.

##### Action subdomain (optimal level)

Action further indicates that a site has responded to and pursued the changes advised by feedback. Wells et al. (2018; p. 228) define their conceptualization of site engagement as “the degree to which [the intervention] was received and acted upon.” This subdomain demonstrates that a site utilizes feedback and pursues improvement, rather than merely reporting on areas of weakness or need. Partaking in learning collaboratives, research networks, or communities of practice may indicate that a site is engaged within the Action subdomain. The Learning Collaborative Model encourages the sharing and application of innovative ideas among members and includes strategies such as learning sessions, webinars or live training, interest circles, coaching, and weekly/monthly electronic newsletters. Despite mixed support (Nadeem et al., 2014), the model may help to facilitate a “culture of process improvement” (Gotham et al., 2023), particularly where multiple practitioners are tackling similar issues and can benefit from a systematic exchange of information and shared solutions (Goodlett et al., 2020).

Communities of practice (CoP) are “groups of people who share a concern or a passion for something they do and learn how to do it better as they interact regularly” (Terry et al., 2020; Wenger, 2011), building on collective learning strategies and increasing organizational capacity to implement the EBP. A CoP might be formed across agencies committed to identifying, adopting, and implementing the most appropriate EBP. CoP members share resources and information while interacting regularly or continuously. Shared experiences allow networks to grow together as well as develop a sense of collective efficacy (Wenger, 2011).

#### Domain 4: Support Exchange

The final domain, *Support Exchange*, includes external funding, facilitation, and other resources made available to the agency from outside sources. This domain centers on intervention sustainment (such as in the EPIS sustainment phase) and is likely to be found in the most engaged organizations that prioritize innovation and sustainment of resources. At the basic-level engagement threshold, the Networks subdomain involves partnerships and associations that the site has developed, potentially including those with the current implementation team or other roles such as employing an expert external facilitator. At the optimal level, the Resources subdomain entails actively seeking and securing external funding and/or assets.

##### Networks subdomain (basic level)

Housed within the Networks subdomain, organizational collaboration and networks (EPIS interconnections, linkages, and relationships) have numerous benefits (Popp et al., 2014), such as improved knowledge exchange and communication (Goodlett et al., 2020; Harris et al., 2012). These valuable partnerships and connections may have been previously fostered and currently relied on, thus indicating that a site has a longer history of site engagement. One example of this support is engaging with an external facilitator, which can itself be considered an intervention to increase site engagement and serve to target different portions of the implementation process (Chaple et al., 2024). The facilitator helps problem-solve by providing additional information and resources for improving implementation or supporting employees’ training about an EBP. The external facilitator can develop a relationship with the internal implementation team to create a supportive environment that improves implementation and keeps the site engaged throughout the process (Ritchie et al., 2020).

Community-academic partnerships (referred to as EPIS bridging factor), and their sustainment, fall squarely in the Network subdomain. Integrating community stakeholders as valued and respected partners is critical in supporting EBP implementation and sustainment (Pellecchia et al., 2018). Such partnerships can address the prevalent research-to-practice gap that implementation science aims to bridge (Drahota et al., 2016). Collaboration with community stakeholders fosters on-the-ground implementation knowledge and promotes the development and curation of practically useful program components (Pellecchia et al., 2018).

##### Resources subdomain (optimal level)

External funding (EPIS bridging factor) is a primary component of the Resources subdomain. Implementing an EBP can have substantial startup costs but typically these costs subsided as the practice becomes routinized. Therefore, despite its demonstrable importance (Jaramillo et al., 2019; Pegg et al., 2021), funding has traditionally been an early barrier to adoption of an EBP and, subsequently, to site engagement. However, some research has shown that sites can adapt and address this barrier by, for example, internally restructuring staffing (Rapp et al., 2010) or acquiring external technical assistance to fund training or quality improvement processes (e.g., the National Institute of Corrections offers these services). As an implementation catalyst, financial resources can promote site engagement such as by funding training and development of staff opportunities (Pegg et al., 2021). Organizations that secure funding or have large budgets in a multisite implementation may become a “sponsor” organization (Margerum, 2001), a role that may hold additional power and decision-making authority.

The *Support Exchange* domain reflects a possible “gold star” engaged organization but is not required for successful implementation. Rather, it can further smooth the implementation process and facilitate sustainment by ensuring that sites implement best practices and have the appropriate time and support to engage. Additionally, funding allocation and *Support Exchange* are often impacted by political pressures from government or outside organizations and, as a result, the lack of funding does not necessarily reflect low site engagement.

#### Structural and organizational context of engagement

We argue that the components of the domains articulated above, and site engagement more generally, should not be considered static or unalterable. Instead, these domains reflect both the structure and strength of the inner and outer contexts, and are influenced by the nature, scope, and connection between situational bridging factors such as those identified in the EPIS framework (Moullin et al., 2019). These factors may include a collaborative versus siloed organizational culture. Prior literature has sought to unpack these structural relationships and how sites interact with others (e.g., Bazzoli et al., 1999). Further, Blau’s (1970) classic review of internal organizational structures revealed that increases in organizational size, differentiation, and specialization affect productivity and engagement; however, these factors can result in enlarged administrative components and costs to coordinate overarching goals and objectives. Consequently, deliberate and continuous efforts to coordinate services across and within agencies and organizations are imperative. Such efforts are also shaped by EPIS determinants of interconnections, interactions, linkages, and relationships (Moullin et al., 2019). An insular agency, for example, may be a hesitant partner largely unwilling to share information.

As the *Collaboration* domain suggests, inter- and intra-organizational partnerships are an important part of site engagement. In a classic study, Gray (1985) acknowledged that inter-organizational collaboration can be especially difficult for organizations representing different sectors with distinct goals and mandates, such as justice and health agencies, where the former is a legal organization with punishment powers and the latter is focused on providing medically related services. In the early stages of inter-agency collaboration, stakeholders should consider the complexity of implementation, determine if they have the authority and expertise to guide or execute change in their organization(s), and ascertain if the benefits outweigh the costs (Aarons et al., 2014b; Gray, 1985). Welsh et al. (2016) examined barriers to inter-agency relationships between community supervision agencies and community treatment providers, finding that a lack of anticipated benefits of collaboration led to decreased engagement among frontline workers.

Finally, Konrad (1996) describes organizational integration as “a process by which two or more entities establish linkages for the purpose of improving outcomes for needy people” (p. 6), with the benefits of increasing accessibility, improving information systems, and cost containment through reduced duplication and inefficiency. Konrad (1996) highlights site differences in levels of coordination and integration that can affect site engagement, such as the nature and extent of formal agreements as well as relationships between agencies and stakeholders, or with researchers.

With these domains and contextual factors in mind, we turn to the context in which our proposed model was developed, the JJ-TRIALS implementation science multisite project. This 36-site project found substantial site differences in the level of involvement and engagement in both the implementation strategies and justice-involved client outcomes. It provides important lessons for assessing site engagement and its measurement.

## Case presentation: juvenile justice translational research on interventions for adolescents in the legal system (JJ-TRIALS)

### Context for the proposed site engagement model

JJ-TRIALS incorporated a multicomponent implementation intervention designed to improve the transition of justice-involved youth through the Behavioral Health Services Cascade (hereafter, Cascade) toward engagement and retention in evidence-based treatment (Belenko et al., 2017; Dennis et al., 2019; Knight et al., 2015). Thirty-six counties were recruited into the study, including one juvenile justice (JJ) community supervision agency (probation departments, juvenile drug courts) and one or two community behavioral health (BH) care providers. The 36 original sites in 7 states received a Core implementation strategy that included a needs assessment and systems mapping exercise; staff training on behavioral health among justice-involved youth, treatment, inter-agency collaboration, data-driven decision making, and goal selection support; and formation of inter-agency workgroups to address agency goals around improving Cascade outcomes. JJ-TRIALS used a multisite cluster randomized design in which matched pairs of sites were randomized to receive either the Core condition, or the Core implementation strategy plus facilitation of local change teams (Enhanced condition) working toward their Cascade goal. JJ-TRIALS researchers evaluated two main research questions and associated hypotheses related to youth outcomes: Does the Core and/or Enhanced condition reduce unmet need by increasing Cascade retention related to screening, assessment, treatment initiation, engagement, and continuing care? Does the addition of the Enhanced condition components further increase the percentage of youth retained in the Cascade relative to the Core components? Other hypotheses related to the impact of JJ-TRIALS on youth recidivism, along with additional staff- and organizational-level hypotheses (Knight et al., 2015). JJ-TRIALS was organized around the EPIS framework (Aarons et al., 2011; Becan et al., 2018; Moullin et al., 2019), capturing the process of implementing EBPs across four successive stages, and the factors that influence movement through those stages. JJ-TRIALS further elaborated the EPIS framework by considering a circular approach to implementation accounting for recursive movement through phases as needed (Becan et al., 2018).

Findings from JJ-TRIALS demonstrated that a complex implementation intervention could be successfully implemented over a multiyear period in a large number of sites across the country.^1^ Assessing youth outcomes along the Cascade in the 36 sites, about 70% of youth were screened for substance use issues, nearly half of youth were identified as being in need of treatment, but only about one-quarter of youth in need were referred to treatment (Belenko et al., 2022; Dennis et al., 2019; Wasserman et al., 2021). However, during the study, referrals increased among youth in need of treatment over baseline, and the Enhanced condition yielded higher referral rates than the Core condition over time (Belenko et al., 2022). In terms of post-referral outcomes, there was an overall increase over time in initiation, engagement, and continuing care of treatment for both the Core and Enhanced group (Knight et al., 2022). That is, penetration through the Cascade increased throughout the experiment relative to baseline. The external facilitation in the Enhanced condition resulted in shorter time to service initiation and greater penetration through the Cascade, compared with the Core condition. Substantial variation in Cascade outcomes was found across sites, as well as variation in the relative impact of the Enhanced condition (Belenko et al., 2022; DeLucca et al., 2022; Knight et al., 2022). These site differences highlight the importance of understanding the inner and outer context factors that can affect the impact of implementation and client outcomes.

In an analysis of one-year recidivism outcomes for 20 JJ-TRIALS sites in five states, Robertson et al. (2023) found that, overall, 31.8% of youth recidivated within one year of initial referral to community supervision. Recidivism for youth in the Enhanced condition sites decreased by 5.6% points from baseline, while increasing by 2% points for youth in the Core sites. Multivariate models revealed that youth in need of substance use treatment and who were placed on formal/more intensive supervision oversight were more likely to be rearrested, and there were significant differences in recidivism across study sites. The interaction of implementation condition by time period was significant. The difference in recidivism between the experimental period and baseline was significant for the Enhanced condition, such that youth in this condition were 9% less likely to recidivate than their Core condition counterparts. Overall, the inconsistency across sites in implementation and Cascade outcomes illustrates the importance of examining variation in site engagement and the development of associated domains and measures.

Similarly, among other factors that led to the development of the SEAMLIS model was a series of analyses conducted using JJ-TRIALS data. During the EPIS phases of the JJ-TRIALS intervention, we observed varying degrees of project involvement among our study sites and even site-specific fluctuations in participation across intervention phases. These observations were confirmed through preliminary analyses of JJ-TRIALS Management Report data and Monthly Site Check-in data, two complementary data sources collected and analyzed as part of the project (Bartkowski et al., 2019). Exploratory factor analysis and cluster analysis both confirm and underscore variability in engagement among sites and across intervention phases. For example, site engagement during the first phase (Exploration) was relatively robust, with a high level of engagement observed among 17 sites, medium engagement among another 17 sites, and low engagement among only 2 sites. Diminished engagement was observed during the second phase (Preparation), with only 5 sites exhibiting a high level of engagement, 7 exhibiting medium engagement, and 23 exhibiting low engagement. The third phase (Implementation) had only 1 highly engaged site; the majority of sites (*n* = 21) demonstrated medium engagement, while the remainder (*n* = 12) manifested a low level of engagement. These results confirmed the need to consider site engagement as an important construct in understanding implementation of various EBPs as part of implementation and process studies, and led to development of SEAMLIS.

### Conceptual and empirical contours of the proposed site engagement model

#### Domain and measurement specification

Once developed, we applied SEAMLIS retrospectively to the JJ-TRIALS experiment. Although JJ-TRIALS did not originally measure site engagement in its design, many of the measures it used (see Knight et al., 2015) can be employed to operationalize SEAMLIS’s domains. Below, we map the set of JJ-TRIAL measures onto the four SEAMLIS domains, and include other general (non-JJ-TRIALS) measures as informed by the literature (see Table 1).

**Table 1:** Proposed variables for site engagement analysis, conceptual model domains

Responsiveness	Collaboration	Feedback	Support Exchange
**Attendance** Orientation Meetings • Leadership Representative ^E^ • Line Staff Representative Workgroup Implementation Intervention Meetings (NA, GSS/DDDM) ^E, P, I, S^ Behavioral Health Training ^P^ (Completed/invited) **Participation** Questions or comments from participants at each meeting attended	**External** Workgroup Initiated Meetings • Number of Months Met ^I, S^ • Number of Meetings ^I, S^ NA focus group indicating level of collaboration ^E^ Action Plans incorporating steps toward collaboration ^P^ **Internal** Staff Survey Collaboration Questions, Administered 1–4 ^E, P, I, S^	**Awareness** Staff Survey Attitudes and Practice Use Questions, Admin 1–4 ^E, P, I, S^ NA focus group indicating awareness of Practice Use ^E^ SFR Meeting ^E^ **Action** MSC Calls indicating Goal Progress ^I, S^	**Networks** Staff Survey Organizational Functioning Questions, Admin 1–4 ^E, P, I, S^ **Resources** Use of Implementation Intervention Tools ^I, S^ Use of Data • Staff Evaluation ^I, S^ • Analyze Youth Records ^I, S^ Service Access, Policy, or Funding Changes along the Service Cascade ^I, S^ WG Turnover ^I, S^ Number of Calls with WG Liaison for Sites in Enhanced Condition ^I, S^ Non-WG Meetings/Calls on Goal Progress

Key: Abbreviations featured in this table are described as follows

GSS = Goal selection support; DDDM = Data-driven decision-making; WG = Workgroup; SFR = Site Feedback Report; NA = Needs Assessment; MSC = Monthly Site Check-in

EPIS Phases: Exploration = E; Preparation = P; Implementation = I; Sustainment = S

Note: For further information on variables and abbreviations, see Knight et al. (2015)

The first domain, *Responsiveness*, refers to Attendance and Participation in internal events, meetings, or trainings held within an agency, and inter-organizational events across agencies or with researchers. JJ-TRIALS offered many examples of applicable variables, such as tracking attendance of leadership and line staff at orientation meetings, goal achievement training (i.e., training in which site workgroup members select a specific goal and learn how to review and use data to inform their decisions), behavioral health training (i.e., online self-paced didactic tutorials and live skills supervision sessions), and monthly meetings/calls with inter-agency workgroups and local change teams (Knight et al., 2015). Participation, or a site’s involvement in an event, is more complex to operationalize and thus less commonly measured but could include site contributions to events and trainings such as the number of questions and comments (Margerum, 2001).

*Collaboration*, the second domain, can represent External Relationships, such as what is received from the researchers and other collaborating agencies to keep the site engaged in EBP implementation, and Internal Relationships, such as support that a site and frontline practitioners receive from their supervisors and other staff. In JJ-TRIALS, staff surveys measured internal and external collaboration. The occurrence of inter-agency meetings, either facilitated by an external research center or arranged at the site’s discretion, can demonstrate the degree to which the agencies wish to collaborate and set action plans to achieve their selected and specified goals. As an example of such meetings, JJ-TRIALS hosted two research center-facilitated events including a needs assessment identifying the degree to which partnering agencies collaborate on service linkage, and goal selection on choosing strategies for addressing collaboration and service gaps. Therefore, the *Collaboration* domain may be best measured both quantitatively through staff surveys and the number of workgroup meetings. Qualitative data can also be collected through focus groups that ask open-ended questions about the degree to which the agency staff feel supported by internal and external collaborators in their implementation efforts.

The third domain, *Feedback*, refers to behaviors that demonstrate a site’s Awareness (knowledge of an issue) and Action (activities addressing service gaps, etc.). Awareness of needs and weaknesses is readily measured through staff surveys and needs assessment focus group tools, both of which were documented in the JJ-TRIALS Site Feedback Report presented to agency leadership. The Action subdomain considers the degree to which a site responds to and makes changes advised through feedback. In JJ-TRIALS, Action was measured using a monthly site check-in call between the research center and site liaison. Those brief calls documented workgroup meetings and other efforts toward addressing the site’s selected goal and action plan. Although not measured in JJ-TRIALS, studies could alternatively administer follow-up assessments that document changes made in response to feedback or if remaining clarifications need attention in achieving goals.

The fourth domain, *Support Exchange*, is represented by two subdomains: Networks, such as leadership support and organizational functioning, and Resources, such as toolkits, external funding, facilitation, and other supports made available to the agency from outside sources. During JJ-TRIALS monthly site check-in calls, agency representatives were asked if the site used tools provided to them to support monitoring goal progress (e.g., goal selection support [GSS] tools and data-driven decision making [DDDM]), policy or funding changes that impacted service linkage and receipt for clients served by their site, and turnover among workgroup members. Turnover is a key issue since it affects the working relationship of actors to further inter-agency relationships at meetings, which are valuable in successfully preparing for and implementing service linkage goals. Additionally, coaching and facilitation activities can be effective measures of *Support Exchange*. In JJ-TRIALS, coaching occurred through site representative-external facilitator meetings for sites in the Enhanced condition. Not measured directly in JJ-TRIALS, external funding dollars procured may also offer a critical metric of *Support Exchange*. Measurement can occur by collecting reports regarding outside funding applications and awards.

## Discussion and conclusions

As we have argued above, site engagement has been understudied as a key component of organizational and client-level outcomes in research studies. Implementation science and related literatures offer critical insights into how scholars might understand this dynamic construct. Four primary domains of site engagement emerge from the existing literature: *Responsiveness*, *Collaboration*, *Feedback*, and *Support Exchange*. We posit that sites involved in an implementation research study can move through these domains hierarchically, moving from minimally engaged to fully engaged in the implementation effort. Against the backdrop of the JJ-TRIALS multisite experiment, which focused on improving substance use treatment outcomes for justice-involved youth (Knight et al., 2015), our site engagement model demonstrates the implications of evaluating the active involvement of sites tasked with delivering services that improve the health of populations in need. Given that we saw decay in site engagement across the four phases of the EPIS model, the construct of site engagement is important to assess implementation and client outcomes. Put plainly, more engaged service providers will likely be more successful in implementing and sustaining the intervention and, ultimately, supporting positive health outcomes for high-risk populations such as youth in the juvenile justice system. We contend that SEAMLIS presents a promising direction by which to support these goals.

### Future directions: potential applications of the site engagement model

While our development of SEAMLIS emerged from implementation strategies with juvenile justice agencies and their behavioral health partners, this model can be readily applied more broadly across both juvenile and adult criminal legal system agencies and associated service providers as a natural next step (Elkington et al., 2021; Knight et al., 2021; Martin et al., 2021). Behavioral health agencies are often connected to other service sectors and could provide a bridge to cross-sector diffusion. In implementation research, site engagement could serve as both a dependent variable (What factors contribute to robust site engagement? ) or an independent variable (How does site engagement facilitate and mediate the achievement of implementation benchmarks or client-level satisfaction with the intervention? ).

It would be valuable to assess whether robust site engagement occurs over the life of a project or merely in the early stages of an intervention, or whether engagement levels at different study phases operate independently and dynamically across those time periods. These are important distinctions that can assist in better understanding the outcomes of a study. Examples of important questions include: Does early engagement as a “strong start” help propel a site through the implementation process? Are some facets of site engagement more influential than others?

While we have presented SEAMLIS as a useful tool in multisite implementation research efforts, the measurement of site engagement is also important to agencies seeking to roll out practice improvements. For these agencies, assessing the robustness of engagement among leadership, supervisors, staff, or external stakeholders may lead to decisions about where to begin these efforts. Once SEAMLIS and relevant measures are more fully developed and tested with client and implementation outcomes, it may be possible to develop interventions to increase positive site engagement and thus improve outcomes.

### Current challenges and promising prospects

Despite its pivotal role in implementation efforts, site engagement remains under-analyzed beyond program adherence and, to date, systematic conceptualization has been lacking. This paper has advanced a holistic but admittedly preliminary conceptualization of site engagement. Our model emerged from a multidisciplinary review of numerous literature bases and our experience with the JJ-TRIALS multisite project. Accordingly, the SEAMLIS framework will need to be assessed using varied implementation projects and settings in order to make any claims of generalizability. Further, empirical tests of the assumptions underlying SEAMLIS are also warranted. Although our model is informed by the literature, quantitative analyses could shed light on whether the domains are parsimonious or interdependent. For example, confirmatory factor analysis could illuminate clustering of the domains and subdomains or the dominance of one domain over the others. Such analyses could also assess site engagement’s influence on both implementation and client outcomes and, for example, whether other factors may be mediating the relationship (e.g., staff behavior).

While we have drawn heavily on JJ-TRIALS and the EPIS framework, other implementation science models and process measures (e.g., Stages of Implementation Completion; Chamberlain et al., 2011) could be potentially integrated for a more granular approach, because our model considers the magnitude and types of agency involvement at any stage of the implementation process. SEAMLIS may also be adapted and then applied across various human service sectors (e.g., primary care, education, nonprofit versus for-profit agencies). We also encourage scholars to expand the possible data points that could operationalize key SEAMLIS constructs or generate new measures, as any single project exhibits empirical limits that are best addressed by using a variety of data sources across diverse projects and study settings.

Among other promising prospects, a SEAMLIS summary index measure can be used flexibly as an independent, dependent, or intervening variable. And, in a practical sense, highly engaged sites could be designated as mentors of their less engaged peer sites to foster engagement. Future research can also explore how site selection may usefully be informed by anticipated involvement (e.g., a site engagement screener or pre-selection agency-project alignment survey) and the complex interplay of engagement facilitators (e.g., leadership buy-in) and barriers (e.g., turnover, outer context issues) throughout implementation. SEAMLIS has implications for the development of interventions to encourage a site’s movement up the ladder of engagement. Further research can illuminate the nuances of site engagement for justice and related agencies in implementation research and offer a structured framework for its measurement.

## Data Availability

No datasets were generated or analysed during the current study.

## Footnotes

1 For additional key JJ-TRIALS findings, see Becan et al. (2020), Dennis et al. (2019), Robertson et al. (2023), Wasserman et al. (2021).
